# Does acute stress induced via cold water immersion increase blood THC concentrations in regular cannabis users?

**DOI:** 10.1007/s00213-025-06833-8

**Published:** 2025-06-14

**Authors:** Danielle McCartney, Jordan Levoux, Rebecca Gordon, Laura Sharman, Katie Walker, Jonathon C. Arnold, Iain S. McGregor

**Affiliations:** 1 Lambert Initiative for Cannabinoid Therapeutics, The University of Sydney, Sydney, New South Wales (NSW) Australia; 2 Brain and Mind Centre, The University of Sydney, Sydney, NSW Australia; 3 School of Psychology, Faculty of Science, The University of Sydney, Sydney, NSW Australia; 4 Sydney Pharmacy School, The University of Sydney, Sydney, NSW Australia

**Keywords:** Cannabinoid, Plasma, Oral fluid, Biomarkers, Drug testing, Cognition, Psychomotor, Impairment, Ice bath

## Abstract

**Rationale:**

Δ^9^-tetrahydrocannabinol (THC), the principal intoxicant in cannabis, is a highly lipophilic substance that is readily stored in fat. Preclinical research suggests that fat-stored THC can be released into circulation under lipolytic conditions, such as stress. However, compelling evidence of this phenomenon is lacking in humans.

**Objectives:**

Here, we investigated the effects of acute stress on blood (plasma) THC (and 11-COOH-THC) concentrations as well as indices of intoxication (e.g., cognitive function, subjective drug effects) in ‘regular’ (i.e., > 3 days/week) cannabis users.

**Methods:**

Fifteen volunteers (*n* = 9 female; cannabis use: 5.0 ± 1.5 days/week) participated in a single-arm (i.e., pre-/post-intervention) trial. The intervention (stressor) was cold water immersion (CWI); specifically, 10 min at ~ 10 °C. Plasma cannabinoid concentrations, cognitive function and subjective drug effects were measured pre-, shortly post- and 2 h post-intervention. Measures of stress (e.g., heart rate [HR], blood pressure [BP], subjective ratings) and lipolysis (i.e., plasma free fatty acid [FFA] and glycerol concentrations) were also obtained.

**Results:**

CWI produced a small but significant stress response, characterised by increased HR and systolic BP and decreased subjective “calmness”. It also increased plasma FFA and glycerol concentrations, albeit modestly. However, neither plasma THC nor 11-COOH-THC concentrations increased from pre- to post-intervention. Cognitive function also remained unchanged, while subjective drug effects were negligible.

**Conclusions:**

Stress induced via brief CWI does not appear to increase blood cannabinoid concentrations or induce intoxication in moderate cannabis users.

## Introduction

Over 200 million people use cannabis each year (United Nations Office on Drugs and Crime 2023). Some, to treat medical conditions such as chronic pain, insomnia, and anxiety– others, to induce euphoria and relaxation (Lintzeris et al. 2022; MacPhail et al. 2022). Indeed, cannabis, specifically its principal phytocannabinoid, *Δ*^*9*^*-tetrahydrocannabinol* (THC), is a well-known intoxicant that causes dose-dependent subjective and physiological changes, including cognitive and psychomotor impairment, in users (McCartney et al. 2021; Wickens et al. 2022).

THC is a highly lipophilic substance with poor aqueous solubility (Banister et al. 2019; Thomas et al. 1990). This means that while most THC (e.g., ~ 80–90%) is excreted within five days of use as hydroxylated and carboxylated metabolites (e.g., 11-OH-THC, 11-COOH-THC), significant quantities are taken up and stored in fat deposits throughout the body (Chayasirisobhon 2020). Indeed, THC has been detected in human fat biopsies 28 days following cannabis use (Johansson et al. 1989). Of course, fat is a biologically active tissue that releases lipids (e.g., free fatty acids [FFAs]) and glycerol into the blood, through a process known as lipolysis, whenever the body requires energy (Edwards and Mohiuddin 2020).

Given that THC is stored in fat, and lipolysis can be triggered by stress; specifically, the release of stress hormones such as adrenocorticotrophic hormone (ACTH), adrenaline and noradrenaline (Edwards and Mohiuddin 2020), scientists have theorised that physical and psychological stressors might increase blood THC concentrations in regular cannabis users (Gunasekaran et al. 2009; Westin et al. 2014; Wong et al. 2013, 2014). This is significant as blood THC concentrations are often used as evidence in medicolegal cases involving highly stressful and/or traumatic events (e.g., motor vehicle accidents, assaults).

This ‘reintoxication hypothesis’ has some scientific support. First, there have been two published cases of anomalously high post-mortem blood THC concentrations (i.e., 90 and 290 ng/mL) in victims of traumatic death (Collins et al. 1997). Second, the administration of ACTH has been shown to increase: (a) the THC concentration of a medium containing rat adipocytes (pre-treated with THC); and (b) blood THC concentrations in rats (pre-treated with THC) (Gunasekaran et al. 2009). Third, stressors such as exercise and food deprivation have been shown to increase blood THC (Gunasekaran et al. 2009) and 11-COOH-THC (Gunasekaran et al. 2009; Wong et al. 2014) concentrations in rats (pre-treated with THC). To date, however, only two studies have investigated the phenomenon in humans.

Wong et al. (2013) measured the plasma THC and 11-COOH-THC concentrations of 14 ‘regular’ (i.e., ≥ 5 days per week) cannabis users before, after, and 2 h after 35 min of cycling at 50% of aerobic capacity (VO_2max_) in either a fed (*n* = 7) or fasted (*n* = 7) state. The study showed that exercise (but not fasting) had a strong (Cohen’s d_z_ = 1.16) and significant (transient) effect to increase plasma THC concentrations. A positive correlation between the exercise-induced change and body mass index (BMI) was also observed, suggesting that individuals with greater fat mass might be more vulnerable to the effect.

Meanwhile, Westin et al. (2014) measured the serum THC and 11-COOH-THC concentrations of six daily cannabis users before and after: (a) 45 min running at 60–75% of age-predicted heart rate (HR) maximum; and (b) 24 h of food deprivation. No “major differences” were said to be observed. However, a considerable proportion (≥ 80%) of participants still had mildly elevated serum THC and 11-COOH-THC concentrations after exercise and fasting, respectively. Further research clarifying whether acute stress increases blood cannabinoid concentrations in regular cannabis users is, therefore, warranted.

The primary aim of this study was to investigate the effects of acute stress on blood (plasma) THC and 11-COOH-THC concentrations in regular cannabis users. The specific stressor employed was *cold water immersion* (CWI), as cold exposure appears to trigger the release of stress hormones (Eimonte et al. 2022) and induce lipolysis (Straat et al. 2022). The secondary aim was to determine whether a stress-induced change in plasma THC concentration was sufficient to: (a) induce mild intoxication, as indexed by subjective reports and objective measures of cognitive function, and (b) elicit positive point-of-care (POC) (e.g., roadside, workplace) oral fluid drug tests. We hypothesised that CWI would increase plasma THC and 11-COOH-THC concentrations, inducing mild intoxication. The likelihood of a stress-induced increase in plasma THC concentration affecting oral fluid THC concentrations was expected to be low.

## Methods

### Study design

A single-arm (i.e., pre-/post-intervention) non-clinical trial was conducted at the University of Sydney. The trial was approved by the University’s Human Research Ethics Committee (2023/269) and conducted in accordance with the Declaration of Helsinki (1983).

### Participants

Eligible volunteers were: (1) aged between 18 and 40 years; (2) ‘regular’ (i.e., *≥* 3 days/week for *≥* 3 months) users of cannabis or THC-containing products (as evidenced by a positive POC urine drug test for 11-COOH-THC); and (3) proficient in English.

The following exclusion criteria applied: (1) hypersensitivity to cold (e.g., Raynaud’s syndrome); (2) recent (i.e., *≤* 3 months prior) involvement in a ‘temperature manipulation’ (i.e., cold stressor) program; (3) BMI < 22.0 kg/m^2^; (4) cardiac or vascular disease (including hypertension); (5) haemophilia; (6) high resting systolic blood pressure (SBP) (i.e., > 140 mmHg); (7) asthma; (8) a neurological disorder or intellectual disability; (9) open wounds; (10) unwilling or unable to adhere to the study procedures (including the standardisation procedures outlined in Sect. 2.5.1); and (11) pregnant or lactating.

### Participant enrolment

Each volunteer completed a short online questionnaire and a telephone interview. Those suitable to continue then scheduled a face-to-face screening. Here, they were informed about the nature and risks of the experimental procedures before providing written informed consent and being assessed for eligibility via self-report, having their height, weight and blood pressure measured, and completing a POC urine drug test (NxStep OnSite Drug Test, DrugCheck^®^, MN, USA). A positive test (i.e., suggesting that urinary 11-COOH-THC concentrations were > 50 ng/mL) was taken as evidence of regular cannabis use. Eligible participants then provided basic demographic information, had their fat mass measured via standard procedures (BC-730 Body Composition Monitor, Tanta^®^, WA, Australia), and practised the cognitive and psychomotor tasks they would complete at their test session.

### Intervention

The intervention (stressor) was CWI. Participants sat in a portable bath (sub-merged to the level of their clavicle) in light clothing or swimwear for a period of 10 min. The bath contained ~ 165 L of cold tap water and ~ 20 kg of ice. The temperature (~ 10 °C) was measured at 2.5-minute intervals throughout CWI (THERM-cert, Brannan^®^, England). This protocol has been shown to trigger the release of hormones (i.e., adrenaline, noradrenaline, cortisol) (Eimonte et al. 2022) that can be expected to induce lipolysis (Straat et al. 2022).

### Test sessions

Each participant completed one test session at the Brain and Mind Centre (BMC).

#### Standardisation procedures

Participants were instructed to: (1) abstain from cannabis and caffeine (> 12 h); (2) abstain from alcohol and other psychoactive substances (> 24 h); (3) spend > 8 h in bed overnight; (4) fast overnight (i.e., > 8 h) (as fasting has been shown to potentiate stress-induced increases in plasma FFA concentrations (Iwayam et al. 2020)); and (5) consume ~ 500 mL of water (i.e., on waking) prior to their test session.

#### Experimental procedures

Participants arrived at the laboratory (in a fasted state) between ~ 07:00–11:00 AM and were asked to indicate whether they complied with each of the standardisation procedures. Compliant participants then provided information about their last cannabis use, re-familiarised themselves with the cognitive and psychomotor tasks, and completed a series of pre-intervention assessments (outlined in Sect. 2.6 ‘Data Collection’) before undergoing CWI. The pre-intervention assessments were repeated shortly post-intervention (i.e., after participants had towel-dried and dressed themselves) and 2 h post-intervention. Individuals were also asked to indicate whether they experienced any “unfavourable signs or symptoms” (adverse events) before leaving the laboratory. Participants remained fasted (i.e., were only permitted to consume plain water) throughout the (~ 3-hour) test session.

### Data collection

#### Primary outcome

Plasma cannabinoid concentrations were measured pre-, ~ 5 min post- and 2 h post-intervention using ultra-high performance liquid chromatography-tandem mass spectrometry (LC-MS/MS) and previously validated methods (Kevin et al. 2021). Samples were analysed in triplicate, with those derived from the same participant analysed on the same plate.

Blood was collected into pre-treated EDTA vacutainers (Beckton Dickson, NJ, USA). The vacutainers were centrifuged at 2500 *g* and 4 °C for 15 min within 30 min of collection and the plasma supernatant was stored at − 80 °C until analysis.

#### Secondary outcomes

The following assessments were conducted pre-, post- and ~ 2 h post-intervention:

- Cognitive and psychomotor function were measured using three computerised tasks: (1) the Digit Symbol Substitution Task (DSST) (1.5 min); (2) the Divided Attention Task (DAT) (~ 4 min); and (3) the Paced Serial Addition Task (PSAT) (~ 4 min). These tasks have previously demonstrated sensitivity to the impairing effects of THC and are detailed elsewhere (Arkell et al. 2019b, 2020).
- Subjective intoxication (i.e., “stoned”, “strength of drug effect”, “euphoric”) was measured using 100 mm visual analog scales (VASs), where zero = “not at all” and 100 = “extremely”.
- A POC oral fluid drug test; specifically, the Dräger DrugTest^®^ 5000 (DT5000), was administered as per the manufacturer’s instructions (Arkell et al. 2019a; McCartney et al. 2022; Suraev et al. 2024). The DT5000 generates a qualitative result (i.e., positive, negative, invalid) for cannabis, cocaine, opiates, benzodiazepines, amphetamines and methamphetamine, and has an adjustable limit of detection (LOD) (i.e., for THC) that was set to 25 ng/mL for the purpose of this experiment.
- Oral fluid THC concentrations were measured using LC-MS/MS and previously published methods (Arkell et al. 2019a; McCartney et al. 2022; Suraev et al. 2024). Samples were analysed in triplicate, with those derived from the same participant analysed on the same plate. Oral fluid was collected using the Quantisal^™^ device (Immunalysis Corporation, CA, USA) and stored at + 4 °C (in the device) until analysis (within 90 days of collection (Scheidweiler et al. 2017).

#### Measures of stress

The following were measured pre-, post- and ~ 2 h post-intervention:

- SBP, diastolic blood pressure (DBP) and heart rate (HR) (in duplicate) using an automated sphygmomanometer (HEM-7121 J, Omron, Kyoto, Japan).
- Subjective stress (i.e., “calm”, “bored”, “nervous”, “excited”) using 100 mm VASs, where zero = “not at all” and 100 = “extremely”.
- Tympanic temperature using a thermometer (DET-1015, Welcare, Hangzhou, China).

The following were measured at 2.5-minute intervals throughout CWI:

- Tympanic temperature (as above).
- HR using a wristwatch (Vivoactive 4 S, Garmin, KN, USA).
- Affective valence using the 11-point Feelings Scale, where − 5 = “feeling very bad” and + 5 = “feeling very good” (Hardy and Rejeski 1989).

#### Measures of lipolysis

Plasma FFA and glycerol concentrations were measured pre-, ~ 5 min post- and 2 h post-intervention using commercial kits (LabAssay NEFA Kit (Cat No. 633–52001), Wako Chemical, Osaka, Japan; Glycerol Colorimetric Assay Kit (Cat No. 10010755), Cayman Chemical, MI, USA). Samples were analysed in triplicate, with those derived from the same participant analysed on the same plate. (Blood was collected, processed, and stored as described in Sect. 2.6.1 ‘Primary Outcome’).

#### Other descriptive measures

Urinary cannabinoid concentrations were measured (in triplicate) using LC-MS/MS and previously published methods (Kevin et al. 2017). A pre-intervention urine sample was collected into a sterile plastic cup and stored at − 80 °C until analysis.

### Statistical analysis

Continuous variables were analysed using linear mixed effects models that included Time (within-subject categorical: pre-, post-, and 2 h post-intervention) as a fixed effect, with Participant as a random effect. Sex (categorical: male and female), Fat Mass (continuous), the Time by Sex interaction, or the Time by Fat Mass interaction was also included as a fixed effect if it reduced the Akaike information criterion (AIC) value of the model. If the residuals were non-normally distributed (Shapiro-Wilk test, *p* < 0.05) or heteroscedastic (Breusch-Pagan test, *p* < 0.05), the dependent variable was square-root transformed and re-analysed (and if unimproved, log transformed). If the variable included zeros, a small positive constant (half the smallest non-zero value) was added prior to transformation. If neither transformation was curative, a gamma generalised linear mixed-effects model (with a log link) was substituted.

Count variables (e.g., certain measures of cognitive function) were also analysed using generalised linear mixed-effects models. These models included the same fixed and random effects as those described above but were fitted to a Poisson distribution (as no data were zero-inflated or over-dispersed).

Two-sided (unadjusted) post hoc comparisons were performed to compare the estimated marginal means if a significant main or interaction effect was observed. Statistical significance was accepted as *p* < 0.05. All values presented in text are estimated marginal means and their 95% confidence intervals (CIs), unless otherwise stated.

All statistical analyses were performed in R version 4.2.0 using the following packages: ‘tidyverse’ (*mutate* function) (Wickham et al. 2019), ‘car’ (*Anova* and *qqp* functions) (Fox and Weisberg 2019), ‘MuMIn’ (*AICc* function) (Bartoń 2022), ‘lme4’ (*lmer* function) (R Core Team 2022), ‘lmerTest’ (Kuznetsova et al. 2017), ‘glmmTMB’ (*glmmTMB* function) (Brooks et al. 2017), ‘emmeans’ (*emmeans* function) (Lenth 2023) and ‘performance’ (*check normality*, *check heteroscedasticity*, *check model* functions) (Lüdecke et al. 2021); ggplot2 (Wickham 2016) and ‘Rmisc’ (Hope 2022) were also used to generate plots.

### Sample size

Wong et al. (2013) found that exercise increased plasma THC concentrations (Cohen’s d_z_ = 1.16) in regular cannabis users. Using a power (1-β) of 0.8, a two-sided α of 0.05, and a more conservative Cohen’s d_z_ of 0.80, we predicted that 15 participants would be required to test the primary hypothesis. A target sample size of 18 was selected to account for attrition.

## Results

### Recruitment and retention

**Fig. 1:**
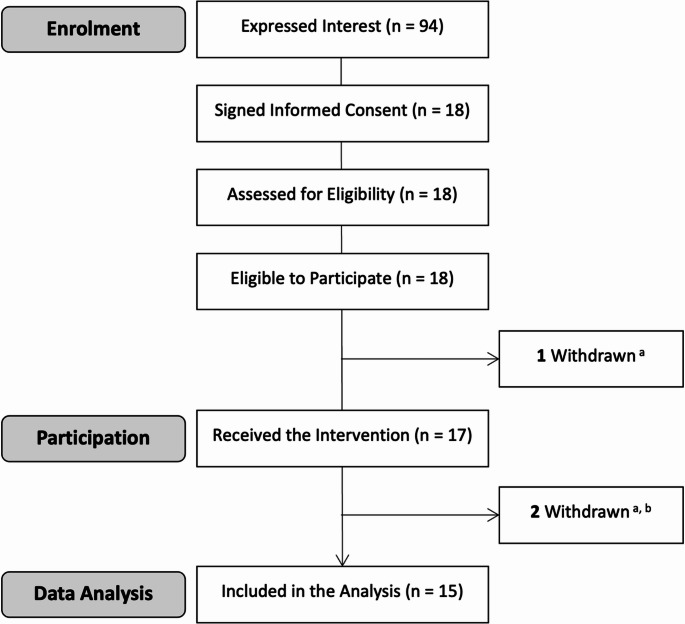
CONSORT diagram. **a**: Recent (< 24 h prior) use of psychoactive substances; **b**: Recent (< 12 h prior) cannabis use (i.e., an oral fluid THC concentration of 33 ng/mL)

Eighteen participants signed informed consent between August 4 and October 10, 2023 (Fig. 1). All were eligible to participate and attended a test session. However, only 17 were exposed to the intervention. The remaining individual was sent home pre-intervention (and excluded from the final sample) after he reported using psychoactive substances (benzodiazepines and amphetamines) < 24 h prior to arrival. A second individual was also excluded after being found to have high oral fluid THC concentrations (i.e., 33 ng/mL), inconsistent with the required 12 h of cannabis abstinence (Lee et al. 2015; Odell et al. 2015). All other participants had oral fluid THC concentrations ≤ 6 ng/mL. Finally, a third participant who (a) returned a positive (pre-intervention) oral fluid drug test for cocaine but (b) was not sent home (as he affirmed that he had not used cocaine < 24 h prior to arrival), was excluded on the basis that residual oral fluid cocaine concentrations seldom exceed 20 ng/mL (Cone and Weddington 1989) (i.e., the LOD on the DT5000 (Gjerde et al. 2018). The 15 remaining participants were included in the final (analytical) sample.

### Participant characteristics

The demographic characteristics of the participant population are summarised in Table 1. Briefly, individuals reported using cannabis an average of 5.0 ± 1.5 days/week (mean ± SD) and ~ 23 [14–40] hours (median [interquartile range, IQR]) prior to their test session. They also had an average BMI and body fat content at the lower end of the ‘overweight’ range (Jeukendrup and Gleeson 2004).

**Table 1:** Baseline characteristics (*n* = 15)

Characteristic	Frequency (*n*), Mean ± SD or Median [IQR]
General:	
Sex (n)	
Male	6 (40%)
Female	9 (60%)
Gender (n)	
Man	6 (40%)
Women	8 (53%)
Non-binary	1 (7%)
Age (years)	29 ± 4
Height (m)	1.71 ± 0.13
Weight (kg)	75 ± 15
BMI (kg/m^2^)	25.1 [22.8–26.4]
Body Fat (%)	
Males	22.4 ± 5.6
Females	32.9 ± 9.1
All	28.7 ± 9.3
Cannabis Use:	
Frequency of use (days/week)	5.0 ± 1.5
Duration of regular use (years)	2.0 [1.0–5.0]
Urinary THC (ng/mL)	1.52 [1.17–1.74]
Urinary 11-OH-THC (ng/mL)	1.15 [0.51–1.90]
Urinary 11-COOH-THC (ng/mL)	26 [10–51]
Perceived potency (n)	
Very strong	4 (27%)
Strong	9 (60%)
Average	2 (13%)
Weak	0
Very weak	0
Usual route of administration (n)	
Inhalation	12 (80%)
Oral ingestion	2 (13%)
Tinctures	1 (7%)
Reason(s) for use (n)	
Recreational purposes	5 (33%)
Medicinal purposes	2 (13%)
Both purposes	8 (53%)

Values are frequency (n), mean (95% CI) or median [IQR], as appropriate (i.e., where data are normal and non-normal, respectively). BMI: Body Mass Index; IQR: Interquartile Range; SD: Standard Deviation

### Intervention characteristics

Zero, 2.5, 5.0, 7.5 and 10 min into CWI, the water temperature was 11.6 [10.9–11.6], 10.5 [10.1–11.1], 10.7 [10.0–11.2], 11.1 [10.3–11.4] and 11.2 [10.6–11.4]°C, respectively (median [IQR]).

### Primary outcomes

Three of the 45 blood samples (2 × post- and 1 × 2 h post-intervention) could not be collected. THC and 11-COOH-THC were present (i.e., above the LOD, 0.1 ng/mL) in all 42 of the remaining samples.

**Fig. 2:**
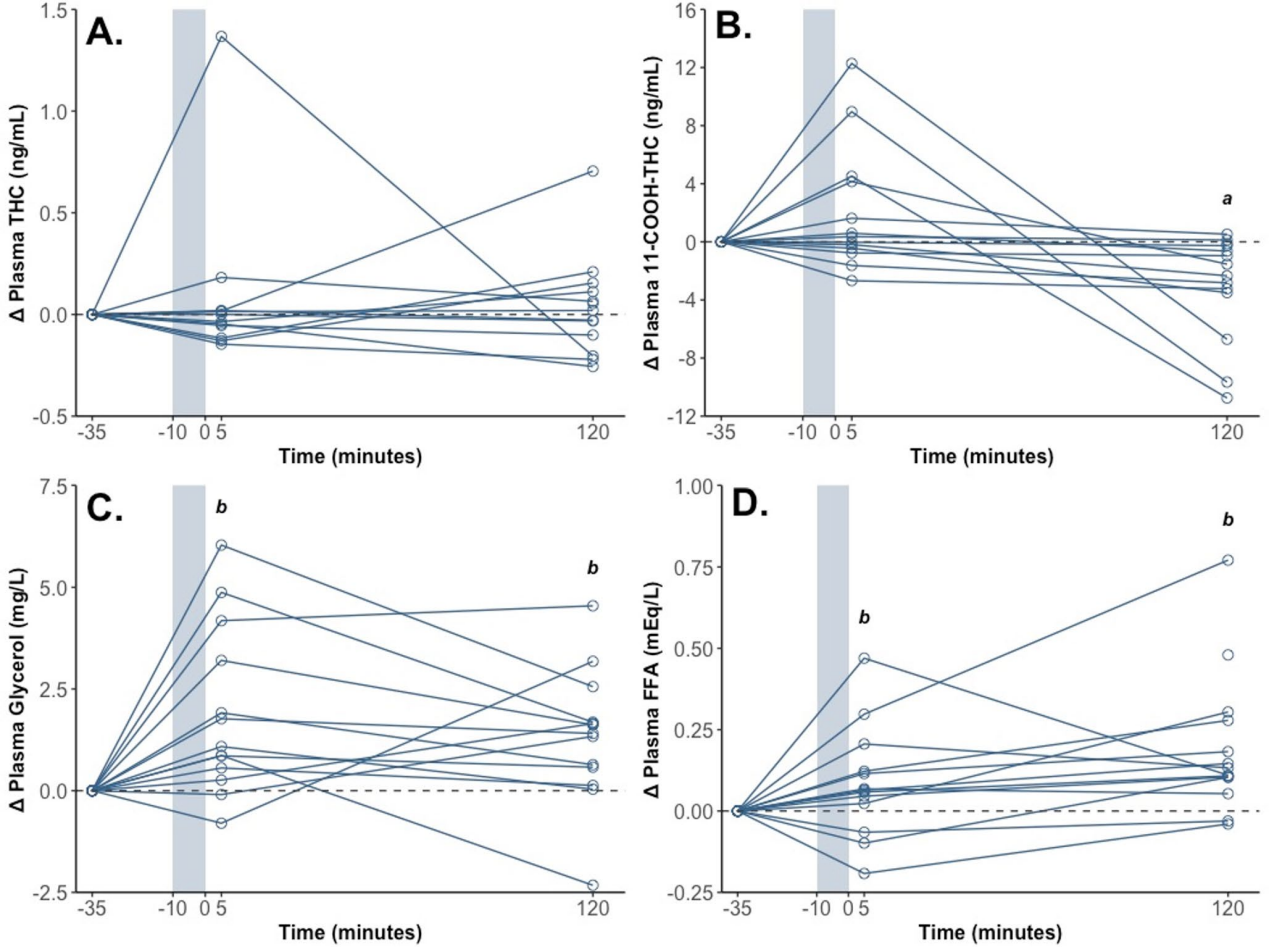
The change in (**A**) Plasma THC, (**B**) Plasma 11-COOH-THC, (**C**) Plasma Glycerol and (**D**) Plasma Free Fatty Acid (FFA) concentrations from pre-intervention (–35 min) to post-intervention (+ 5 min) and 2 h post-intervention (+ 120 min). The shaded region represents the intervention (Cold Water Immersion). Each line represents one individual participant. a: Differs from pre- and post-intervention (*p* < 0.05); b: Differs from pre-intervention (*p* < 0.05) (as raw values)

Plasma THC concentration (Table 2; Fig. 2A) did not demonstrate a significant main effect of Time (Table 3). However, plasma 11-COOH-THC concentration (Table 2; Fig. 2B) did (Table 3). Post hoc comparisons showed that concentrations were higher pre-intervention (16 (5, 32) ng/mL, *p* = 0.002) and post-intervention (17 (6, 34) ng/mL, *p* < 0.001) than 2 h post-intervention (13 (4, 29) ng/mL) – but did not differ pre- and post-intervention (*p* = 0.215).

**Table 2:** Descriptive statistics

	Baseline	Post-Intervention	2 h Post-Intervention
Primary Outcomes ^a, b^			
Plasma Cannabinoid Concentrations			
Plasma THC (ng/mL)	0.70 [0.51–1.62]	0.63 [0.46–1.69]	0.59 [0.35–1.51]
Plasma 11-COOH-THC (ng/mL)	8.2 [4.9–19.4]	7.8 [5.9–24.8]	6.5 [3.9–18.0]
Secondary Outcomes ^c^			
Digit Symbol Substitution Task			
Correct Responses (n)	43.5 [39.5–49.0]	45.5 [39.8–50.8]	45.0 [43.5–48.0]
Attempts (n)	45.5 [42.0–50.8]	46.0 [43.0–50.8]	47.0 [45.5–50.5]
Response Accuracy (%)	97.3 [92.4–99.6]	99.0 [97.7–100]	97.8 [93.9–99.2]
Divided Attention Task			
Hits (n)	24.0 [23.0–24.0]	23.0 [22.0–24.0]	23.0 [21.0–23.0]
Response Time (ms)	920 [778–1150]	1033 [911–1188]	1081 [885–1307]
Tracking Error (pixels)	11.7 ± 3.5	11.4 ± 2.5	12.3 ± 3.0
Paced Serial Addition Task			
Correct Responses (n)	47.8 ± 8.3	44.9 ± 8.8	44.3 ± 9.6
Response Time (ms)	1367 ± 184	1321 ± 130	1334 ± 135
Subjective Intoxication			
Stoned _(0–100 mm)_	0 [0–0]	1 [0–8]	1 [0–5]
Drug Effect _(0–100 mm)_	0 [0–0]	2 [0–6]	1 [0–3]
Euphoric _(0–100 mm)_	0 [0–8]	5 [0–24]	2 [1–16]
Positive Oral Fluid Drug Tests (n)	1	0	1
Measures of Stress ^d^			
Subjective Stress			
Calm _(0–100 mm)_	76 ± 19	51 ± 24	78 ± 14
Bored _(0–100 mm)_	18 [7–45]	15 [3–44]	22 [7–60]
Nervous _(0–100 mm)_	32 [17–59]	27 [5–56]	5 [1–15]
Excited _(0–100 mm)_	57 [50–67]	26 [14–55]	19 [8–50]
SBP (mmHg)	117 ± 9	122 ± 15	117 ± 11
DBP (mmHg)	78 ± 7	81 ± 12	78 ± 10
Measures of Lipolysis			
Plasma Glycerol Concentrations (mg/L)	4.17 ± 3.11	5.73 ± 3.44	5.00 ± 3.00
Plasma FFA Concentrations (mEq/L)	0.28 ± 0.15	0.33 ± 0.16	0.44 ± 0.22

Values are frequency (n), mean ± standard deviation, or median [IQR], as appropriate (i.e., where data are normal and non-normal, respectively). DBP: Diastolic Blood Pressure; FFA: Free Fatty Acids; SBP: Systolic Blood Pressure. a: Plasma 11-OH-THC, CBD, 7-COOH-CBD, 7-OH-CBD, 6-OH-CBD, CBG and CBDV concentrations are not presented as these analytes were seldom detected; b: Plasma 7-OH-CBD, 6-OH-CBD, CBG and CBDV concentrations are not presented as these analytes were never detected; c: Oral fluid THC concentration is not presented as THC was only occasionally detected; d: Heart rate and tympanic temperature are reported in Fig. 4

**Table 3:** The probability (*p*) values for the fixed effects included in each statistical analysis

		Fixed Effects					
		Time		Sex		Time$\:\times\:$Sex	
	Model	F or χ^2^	***p*** value	F or χ^2^	***p*** value	F or χ^2^	***p*** value
Primary Outcomes:							
Plasma THC	Gamma	χ^2^_2_ = 0.103	0.950	-	-	-	-
Plasma 11-COOH-THC	SQRT	F_[2, 25.0]_ = 12.1	**< 0.001**	F_[1, 13.0]_ = 0.300	0.594	-	-
Secondary Outcomes							
DSST Correct Responses	Poisson	χ^2^_2_ = 0.548	0.760	-	-	-	-
DSST Attempts	Poisson	χ^2^_2_ = 0.554	0.758	-	-	-	-
DSST Response Accuracy	Gamma	χ^2^_2_ = 2.19	0.335	-	-	-	-
DAT Hits	Poisson	χ^2^_2_ = 0.531	0.767	-	-	-	-
DAT Response Time	SQRT	F_[2, 26]_ = 0.915	0.413	F_[1, 13]_ = 2.13	0.168	F_[2, 26]_ = 0.778	0.470
DAT Tracking Error	Standard	F_[2, 26]_ = 1.82	0.182	F_[1, 12]_ = 5.27	**0.041**	-	-
PSAT Correct Responses	Poisson	χ^2^_2_ = 1.14	0.565	-	-	-	-
PSAT Response Time	SQRT	F_[2, 25.2]_ = 0.798	0.461	F_[1, 13.1]_ = 0.448	0.497	F_[2, 25.2]_ = 2.21	0.130
Stoned ^a^	Gamma	χ^2^_2_ = 12.9	**0.002**	-	-	-	-
Drug Effect ^a^	LOG	F_[2, 28]_ = 7.72	**0.002**	-	-	-	-
Euphoric ^a^	SQRT	F_[2, 28]_ = 3.06	0.063	-	-	-	-
Measures of Stress							
Calm	Gamma	χ^2^_2_ = 11.3	**0.003**	-	-	-	-
Bored ^a^	SQRT	F_[2, 26]_ = 0.221	0.803	F_[1, 13]_ = 0.057	0.815	F_[2, 26]_ = 1.548	0.232
Nervous ^a^	SQRT	F_[2, 26]_ = 5.06	**0.014**	F_[1, 13]_ = 0.993	0.337	F_[2, 26]_ = 0.333	0.720
Excited ^a^	SQRT	F_[2, 28]_ = 10.5	**< 0.001**	-	-	-	-
SBP	SQRT	F_[2, 28]_ = 4.47	**0.021**	F_[1, 13]_ = 16.1	**0.001**	-	-
DBP	Standard	F_[2, 26]_ = 1.67	0.208	F_[1, 13]_ = 1.46	0.248	F_[2, 26]_ = 1.02	0.376
HR	SQRT	F_[6, 80.4]_ = 8.58	**< 0.001**	-	-	-	-
Tympanic Temperature	Gamma	χ^2^_6_ = 25.0	**< 0.001**	χ^2^_1_ = 2.69	0.101	-	-
Measures of Lipolysis							
Plasma Glycerol ^b^	Standard	F_[2, 24.1]_ = 5.90	**0.008**	-	-	-	-
Plasma FFA	LOG	F_[2, 24.1]_ = 8.88	**0.001**	-	-	-	-

-: Not required; DAT: Divided Attention Task; DBP: Diastolic Blood Pressure; DSST: Digit Symbol Substitution Task; FFA: Free Fatty Acids; HR: Heart Rate; LOG: Dependent variable was log-transformed; PSAT: Paced Serial Addition Task; SBP: Systolic Blood Pressure; SQRT: Dependent variable was square root-transformed. a: A small positive constant (half the smallest non-zero value) was added prior to transformation to remove zeros; b: ‘Plate’ (Plate 1 vs. Plate 2) was a fixed effect in this analysis (based on visual inspection of the data) (F_[1, 11,5]_ = 28.0; *p* < 0.001). Fat Mass and the Time by Fat Mass interaction were not included in any of the models

11-OH-THC, CBD and 7-COOH-CBD were only detected (i.e., > 0.1 ng/mL) in a subset of samples (i.e., < 4 participants), while 6-OH-CBD, 7-OH-CBD, CBG and CBDV were not detected (i.e., > 0.1 ng/mL) in any samples. Hence, these data were not subjected to analysis.

### Secondary outcomes

#### Cognitive and psychomotor function

No measures of cognitive or psychomotor function demonstrated significant main effects of Time (Tables 2 and 3).

#### Subjective intoxication

Both “stoned” and “strength of drug effect” demonstrated significant main effects of Time (Tables 2 and 3). Post hoc comparisons showed that ratings were higher post-intervention (stoned: 3.2 (1.6, 6.5) mm; drug effect: 2.2 (1.1, 4.2) mm) than pre-intervention (stoned: 1.0 (0.5, 1.9) mm, *p* = 0.002; drug effect: 0.6 (0.3, 1.1) mm, *p <* 0.001) and higher 2 h post-intervention (stoned: 2.8 (1.4, 5.4) mm, *p* = 0.002; drug effect: 1.5 (0.8, 2.3) mm) than pre-intervention (*p* = 0.010). “Euphoric” did not demonstrate a significant main effect of Time (Tables 2 and 3).

#### Oral fluid THC concentrations

THC was only present (i.e., above the LOD, 0.5 ng/mL) in 17 of the 45 oral fluid samples collected (i.e., 7 participants). Hence, these data were not subjected to analysis.

#### Oral fluid drug tests

Two POC oral fluid drug tests were THC-positive: one pre- and the other 2 h post-intervention. The corresponding oral fluid THC concentrations were 1.9 and 4.1 ng/mL respectively. The 43 remaining tests were negative (all substances).

### Measures of stress

**Fig. 3:**
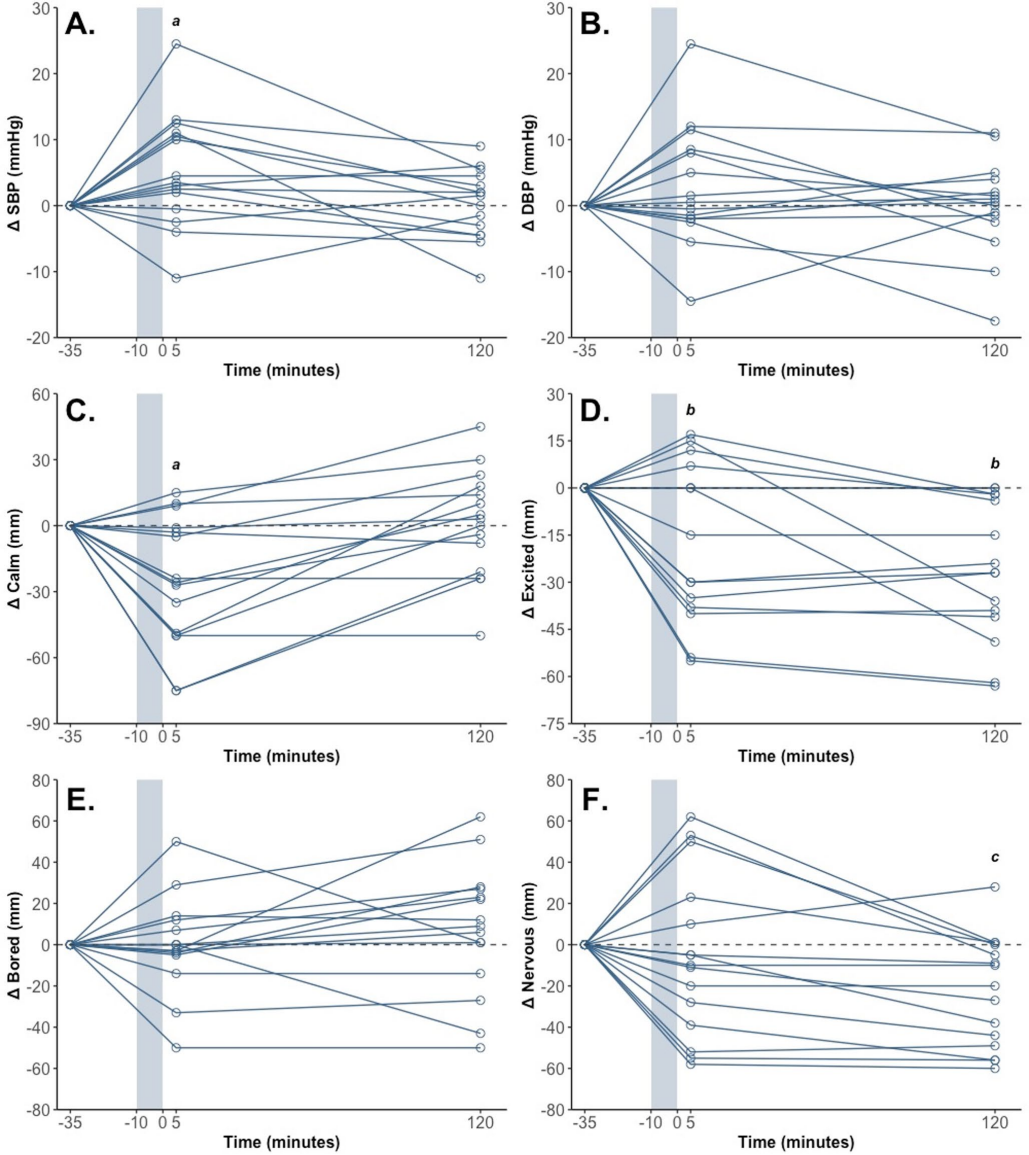
The change in (**A**) Systolic Blood Pressure (SBP), (**B**) Diastolic Blood Pressure (DBP), (**C**) “Calm”, (D) “Excited”, (**E**) “Bored” and (**F**) “Nervous” from pre-intervention (–35 min) to post-intervention (+ 5 min) and 2 h post-intervention (+ 120 min). The shaded region represents the intervention (Cold Water Immersion). Each line represents one individual participant. a: Differs from pre-intervention and 2 h post-intervention (*p*’s < 0.05); b: Differs from pre-intervention (*p* < 0.05); c: Differs from pre-intervention and post-intervention (*p*’s < 0.05) (as raw values)

**Fig. 4:**
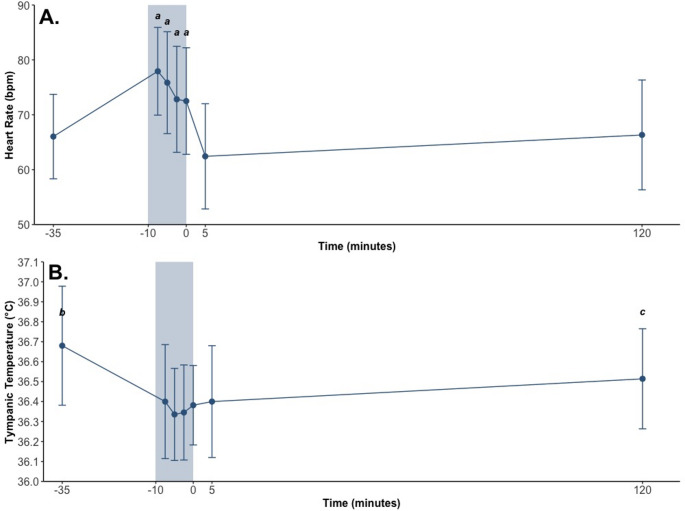
(**A**) Heart Rate and (**B**) Tympanic Temperature pre-intervention (–35 min), post-intervention (+ 5 min), 2 h post-intervention (+ 120 min) and at 2.5-minute intervals throughout the intervention. The shaded region represents the intervention (Cold Water Immersion [CWI]). Values are Mean ± SD. a: Differs from pre-, post- and 2-hours post-intervention (*p*’s < 0.05); b: Differs from all other timepoints (*p*’s < 0.05); c: Differs from pre-intervention and 10 min into CWI (*p*’s < 0.05) (as raw values)

“Calm”, “nervous”, “excited”, SBP, HR and tympanic temperature all demonstrated significant main effects of Time (Tables 2 and 3; Figs. 3 and 4). Post hoc comparisons showed that:

- Ratings of “calm” were lower post-intervention (51 (42, 62) mm) than pre-intervention (77 (63, 94) mm; *p* = 0.004) and 2 h post-intervention (77 (63, 95) mm; *p* = 0.003);
- SBP was higher post-intervention (123 (119, 128) mmHg) than pre-intervention (119 (114, 123) mmHg; *p* = 0.014) and 2 h post-intervention (119 (114, 123) mmHg; *p* = 0.017);
- Ratings of “nervous” were higher pre-intervention (30 (17, 48) mm; *p* = 0.004) and post-intervention (22 (11, 37) mm; *p* = 0.041) than 2 h post-intervention (8 (2, 18) mm);
- Ratings of “excited” were higher pre-intervention (48 (32, 68) mm) than post-intervention (31 (18, 46) mm; *p* = 0.010) and 2 h post-intervention (22 (11, 34) mm; *p* < 0.001);
- HR (Fig. 4A) was higher throughout CWI than pre-, post- and 2 h post-intervention (all *p*’s < 0.05); and.
- Tympanic temperature (Fig. 4B) was lower (a) throughout CWI, post-intervention and 2 h post-intervention than pre-intervention and (b) 10 min into CWI than 2 h post-intervention (all *p*’s < 0.05).

Neither “bored” nor DBP demonstrated significant main effects of Time (Tables 2 and 3).

At 2.5, 5.0, 7.5 and 10 min into CWI, participants’ rated their affective state − 1 [−2–0], −1 [−2–1], 0 [−1–0] and 0 [−1–2], respectively (median [IQR]).

### Measures of lipolysis

Glycerol and FFA were present in all 42 blood samples. (Note: ‘Plate’ (categorical: Plate 1 and Plate 2) was included as a fixed effect in the analysis of plasma glycerol concentrations as differences were readily apparent across plates and its inclusion reduced the AIC value of the model).

Both plasma glycerol concentration and plasma FFA concentration (Table 2; Fig. 2) demonstrated significant main effects of Time (Table 3). Post hoc comparisons showed that concentrations were higher post-intervention (glycerol: 6.11 (4.92, 7.31) mg/L; *p* = 0.003; FFA: 0.33 (0.24, 0.44) mEq/L; *p* = 0.032) and 2 h post-intervention (glycerol: 5.54 (4.37, 6.71) mg/L; *p* = 0.029; FFA: 0.41 (0.31, 0.55) mEq/L; *p* < 0.001) than pre-intervention (glycerol: 4.34 (3.19, 5.48) mg/L; FFA: 0.24 (0.18, 0.32) mEq/L). Plasma FFA concentrations also tended to be higher 2 h post-intervention than post-intervention (*p* = 0.084).

### Adverse events

Two AEs (both mild) were reported: ‘tingling skin’ (post-intervention) and persistent cold (2 h post-intervention).

## Discussion

This study investigated the effects of CWI on plasma THC and 11-COOH-THC concentrations in regular cannabis users. Indeed, CWI was expected to induce stress, thereby triggering lipolysis and, with it, the release of fat-stored THC, leading to an increase in circulating cannabinoid concentrations. As anticipated, CWI appeared to induce ‘stress’, increasing HR and SBP and decreasing tympanic temperature and subjective ratings of “calm”. This stress, in turn, appeared to trigger lipolysis, with plasma FFA and glycerol concentrations increasing from pre- to post-intervention. Contrary to our hypothesis, however, no significant stress-induced changes in plasma THC or 11-COOH-THC concentrations were observed.

Our findings contrast those of Wong et al. (2013) and, to a certain extent, Westin et al. (2014). Indeed, the former found that 35 min of cycling at 50% VO_2max_ increased plasma THC concentrations in regular cannabis users, while the latter found that running at 60–75% of age-predicted HR maximum increased serum THC concentrations in *most* (i.e., 4 out of 5) participants. This discrepancy could be due to differences in the intensity of the stressors employed. On average, CWI increased participants’ HRs to ~ 80 bpm, or ~ 40% of age-predicted HR maximum, whereas exercising at 50% VO_2max_ could be expected to increase HR to ~ 130 bpm, or ~ 70% of age-predicted HR maximum (assuming an age of 30 (Swain et al. 1994)). The exercise interventions were also longer in duration. These differences are notable as they have the potential to impact (Horowitz and Klein 2000; Rodahl et al. 1964), and appear to have impacted, the degree of lipolysis observed. Indeed, while CWI increased participants’ plasma FFA and glycerol concentrations by 18% and 37%, respectively, exercise (in Wong et al. (2013) increased them by a more substantial ~ 110% and 190%. It could, therefore, be that our stressor was not prolonged and/or intense enough to induce the degree of lipolysis required to increase plasma THC concentrations (bearing in mind that *too much* sympathetic activation can also blunt lipolysis (Horowitz and Klein 2000; Rodahl et al. 1964)). This suggests that, in order for a ‘real world’ stressor (e.g., motor vehicle accident, assault) to increase blood THC concentrations, it must also be sufficiently prolonged and/or intense. That said, other factors might also influence whether such an effect is observed.

One such factor could be the extent (or ‘heaviness’) of cannabis use (e.g., frequency of use, amount consumed). The participants studied here reported using cannabis an average of five, and a minimum of three, days per week. Whereas the participants in Wong et al. (2013) and Westin et al. (2014) reported using cannabis a minimum of five and seven days per week, respectively. In fact, those in Westin et al. (2014) reported consuming an average of ~ 18.8 g/week. This heavier cannabis use could have increased participants’ ‘cannabinoid reserves’ and, therefore, likelihood of experiencing a stress-induced change in blood THC concentration. The fact that Westin et al. (2014)’s observations were less compelling than Wong et al. (2013) is difficult to interpret. Nevertheless, It may be worth noting that while the latter required participants to abstain from cannabis use overnight (i.e., pre-stressor), the former required them to abstain for between *three and five days*. Most of the participants studied here (N = 8; 57%) likewise reported abstaining for > 24 hours (N = 3, > 48 hours). It could, therefore, be that such an effect is more likely to occur following ‘semi-recent’ (e.g., < 12 hours prior) cannabis use. However, further research is required to confirm as much.

While neither plasma THC nor 11-COOH-THC concentrations differed pre- *versus* post-intervention, plasma 11-COOH-THC concentrations did fluctuate across time. Indeed, concentrations were found to be higher pre- and post-intervention than 2 h post-intervention. This result is difficult to interpret. However, one possible explanation is that ‘time’ (e.g., ongoing metabolism and excretion) had a subtle effect to decrease plasma 11-COOH-THC concentrations. This effect also appears to be present in Wong et al. (2013), where 12 of the 14 participants were found to have higher plasma 11-COOH-THC concentrations pre- than 2 h post-intervention. It is important to note that while 11-COOH-THC is unlikely to be stored in fat (Lust et al. 2022), any THC that is released from fat could be metabolised to 11-COOH-THC, causing a spike in its concentrations.

As CWI did not increase plasma THC concentrations, it is unsurprising that cognitive and psychomotor function remained unchanged. Further research is, therefore, still required to determine whether a stress-induced increase in blood THC concentration is sufficient to impair cognitive and psychomotor function. Given that studies of this nature require regular cannabis users, and regular cannabis users appear to experience less THC-induced impairment than occasional cannabis users (i.e., likely due to the development of ‘tolerance’) (Colizzi and Bhattacharyya 2018; McCartney et al. 2021), such an effect could be difficult to detect (or absent). Researchers should also be mindful that exercise (which appears to be a suitable stressor) is known to enhance cognitive function (Chang et al. 2012), and that its beneficial effects can ‘overpower’ the detrimental effects of other stimuli (e.g., dehydration (Irwin et al. 2018; McCartney et al. 2019)). Thus, in future studies, it might be appropriate to test the specific hypothesis that exercise is *less* cognitively enhancing in regular cannabis users than it is in non-cannabis users (i.e., as opposed to detrimental).

Perhaps more surprising is the fact that CWI increased subjective feelings of intoxication (e.g., “stoned”) without impacting plasma THC concentrations. This finding could relate to the fact that CWI has been reported to improve mood, increase positive affect, and decrease negative affect (Kelly and Bird 2022; Yankouskaya et al. 2023). However, as participants were unblinded (and fully aware of the study’s purpose), it is impossible to rule out a placebo effect. In either case, the impact was extremely small (i.e., < 2 out of 100 mm on the VAS).

Finally, it is important to note that, although worth confirming, the likelihood of a stress-induced increase in blood THC concentration affecting oral fluid THC concentrations and eliciting positive POC drug tests was always expected to be low. This is because THC appears unable to move from blood into oral fluid (Huestis 2007). Indeed, the THC that is present in oral fluid is thought to originate exclusively from contamination of the oral mucosa (i.e., *during* inhalation or oral ingestion) (Huestis 2007). This, combined with the fact that two positive POC drug tests were observed at oral fluid THC concentrations < 25 ng/mL (i.e., the LOD on the DT5000), suggests these tests should be used and interpreted with caution.

This study is limited by the fact that it was not randomised, controlled, or blinded. It is particularly important to note that, without a control group (i.e., no CWI), we cannot disentangle the effects of the intervention from those of ‘time’. Thus, we cannot be certain that the observed changes in plasma FFA and glycerol concentrations were due to CWI alone; indeed, these could, in part, have been due to fasting (Edwards and Mohiuddin 2020). That said, as the primary purpose of CWI was to induce lipolysis (rather than specifically study its effects on blood cannabinoid concentrations), this distinction has limited bearing on the study’s findings. It is also worth noting that time alone would have been unlikely to explain a post-intervention increase in blood cannabinoid concentrations (had this been observed).

## Conclusion

Stress induced via brief CWI does not appear to increase blood THC or 11-COOH-THC concentrations in moderate cannabis users. Further research investigating the effects of different types and intensities of stress in different cannabis users (e.g., intermittent, heavy) following different periods of cannabis abstinence is required to determine if, and if so, when ‘reintoxication’ might occur– and whether it is of any clinical significance in humans.

## Data Availability

The deidentified participant data are available from Dr Danielle McCartney upon reasonable request (danielle.mccartney@sydney.edu.au).
